# Cross-sectional association of volume, blood pressures, and aortic stiffness with left ventricular mass in incident hemodialysis patients: the Predictors of Arrhythmic and Cardiovascular Risk in End-Stage Renal Disease (PACE) study

**DOI:** 10.1186/s12882-015-0131-4

**Published:** 2015-08-07

**Authors:** Esther D. Kim, Stephen M. Sozio, Michelle M. Estrella, Bernard G. Jaar, Tariq Shafi, Lucy A. Meoni, Wen Hong Linda Kao, Joao A. C. Lima, Rulan S. Parekh

**Affiliations:** 1 Division of Epidemiology, Dalla Lana School of Public Health, University of Toronto, Toronto, Ontario Canada; 2 Department of Medicine, School of Medicine, Johns Hopkins University, Baltimore, Maryland USA; 3 Welch Center for Prevention, Epidemiology and Clinical Research, Johns Hopkins University, Baltimore, Maryland USA; 4 Nephrology Center of Maryland, Baltimore, Maryland USA; 5 Department of Biostatistics, Bloomberg School of Public Health, Johns Hopkins University, Baltimore, Maryland USA; 6 Department of Epidemiology, Bloomberg School of Public Health, Johns Hopkins University, Baltimore, Maryland USA; 7 Department of Pediatrics and Medicine, School of Medicine, University of Toronto, Toronto, Ontario Canada; 8 Hospital for Sick Children, University Health Network and University of Toronto, Toronto, ON Canada

## Abstract

**Background:**

Higher left ventricular mass (LV) strongly predicts cardiovascular mortality in hemodialysis patients. Although several parameters of preload and afterload have been associated with higher LV mass, whether these parameters independently predict LV mass, remains unclear.

**Methods:**

This study examined a cohort of 391 adults with incident hemodialysis enrolled in the Predictors of Arrhythmic and Cardiovascular Risk in End Stage Renal Disease (PACE) study. The main exposures were systolic and diastolic blood pressure (BP), pulse pressure, arterial stiffness by pulse wave velocity (PWV), volume status estimated by pulmonary pressures using echocardiogram and intradialytic weight gain. The primary outcome was baseline left ventricular mass index (LVMI).

**Results:**

Each systolic, diastolic blood, and pulse pressure measurement was significantly associated with LVMI by linear regression regardless of dialysis unit BP or non-dialysis day BP measurements. Adjusting for cardiovascular confounders, every 10 mmHg increase in systolic or diastolic BP was significantly associated with higher LVMI (SBP β = 7.26, 95 % CI: 4.30, 10.23; DBP β = 10.05, 95 % CI: 5.06, 15.04), and increased pulse pressure was also associated with higher LVMI (β = 0.71, 95 % CI: 0.29, 1.13). Intradialytic weight gain was also associated with higher LVMI but attenuated effects after adjustment (β = 3.25, 95 % CI: 0.67, 5.83). PWV and pulmonary pressures were not associated with LVMI after multivariable adjustment (β = 0.19, 95 % CI: −1.14, 1.79; and β = 0.10, 95 % CI: −0.51, 0.70, respectively). Simultaneously adjusting for all main exposures demonstrated that higher BP was independently associated with higher LVMI (SBP β = 5.64, 95 % CI: 2.78, 8.49; DBP β = 7.29, 95 % CI: 2.26, 12.31, for every 10 mmHg increase in BP).

**Conclusions:**

Among a younger and incident hemodialysis population, higher systolic, diastolic, or pulse pressure, regardless of timing with dialysis, is most associated with higher LV mass. Future studies should consider the use of various BP measures in examining the impact of BP on LVM and cardiovascular disease. Findings from such studies could suggest that high BP should be more aggressively treated to promote LVH regression in incident hemodialysis patients.

**Electronic supplementary material:**

The online version of this article (doi:10.1186/s12882-015-0131-4) contains supplementary material, which is available to authorized users.

## Background

Left ventricular hypertrophy (LVH) is the most frequently observed cardiac abnormality in end-stage renal disease (ESRD), and strongly predicts cardiovascular mortality in hemodialysis patients [1]. Despite past findings demonstrating associations between various cardiac parameters and LVH [2–4], independent, modifiable, and clinically relevant predictors of left ventricular mass (LVM) in ESRD patients remain difficult to define. Clinical trials have shown that treatment of anemia and baseline factors such as cardiac troponin T, C-reactive protein, IL-6, and serum albumin are not associated with LV progression over follow-up [5]. Furthermore, the relative importance of various modifiable risk factors in predicting LVH in incident dialysis patients (i.e. patients transitioning from chronic kidney disease [CKD] to ESRD), in particular, remains unclear, as the majority of studies have included prevalent dialysis patients, predominantly Europeans, with potential survivor and selection bias as well as small sample sizes limiting analyses. African-Americans have also not been as extensively studied despite the high risk of hypertension and LVH prior to ESRD in this population.

Hypertension is a major risk factor for LVH, and the role of blood pressure (BP) in the progression of LVM and mortality of patients receiving hemodialysis has been well documented [6]. The timing of BP measurements, however, has been controversial in dialysis patients, as BP levels are highly variable and BP measurements at a single time point may not be a reliable estimate of the arterial pressure load [7, 8].

Other factors associated with LVH are arterial stiffness and volume overload. Aortic pulse wave velocity (PWV) predicts cardiovascular disease and mortality in patients with hypertension and ESRD [9–11], and increased arterial stiffness measured by PWV is also associated with an increase in LVH or LVM in cross-sectional analyses of prevalent dialysis patients [2, 12]. Volume overload is closely associated with hypertension due to the positive sodium balance, which leads to hypertension and increased extracellular volume [13]. Studies have suggested that controlling for circulatory volume can decrease BP, contribute to LVH regression, and improve survival [13, 14].

These parameters of arterial compliance and ventricular volume [2, 15], pre- and post-hemodialysis systolic blood pressure (SBP) [3, 4], and intradialytic weight gain (IDWG) [13, 14] have been implicated with higher left ventricular mass index (LVMI). However, these associations have not been consistently associated in studies likely due to the complicated interrelationship among these factors. Furthermore, there are only a few studies that incorporate all these vascular and volume measures and determine the independent associations of vascular, arterial, and volume measures with LVM, as most studies with smaller sample sizes demonstrate that a single factor is associated with LVMI. However, clinically, it is important to understand which factor should be of most importance. This study therefore examines the relevant importance and the independent roles of blood pressure, arterial stiffness, and volume in predicting LVH using modifiable risk factors that are clinically relevant and available in a large prospective cohort of incident hemodialysis participants.

## Methods

### Study participants

We conducted cross-sectional analyses using data from participants enrolled in the Predictors of Arrhythmic and Cardiovascular Risk in End Stage Renal Disease (PACE) study, which enrolled 402 adult incident hemodialysis patients who completed a baseline cardiovascular study visit. The methods of the PACE study have been described in detail previously [16]. Briefly, PACE is a prospective cohort study in the greater Baltimore area, which enrolled participants from November 2008 to August 2012 from 27 outpatient dialysis units. Eligible participants were hemodialysis patients aged 18 years or older within six months of hemodialysis initiation, and able to provide informed consent. Participants were excluded if they were on home dialysis, had active cancer other than nonmelanoma skin cancer, had a pacemaker/automatic implantable cardiac defibrillator, had detectable atrial fibrillation at the time of echocardiogram, were pregnant, were nursing mothers or were non-English speaking patients. In this analysis, participants without LVMI measurements were further excluded.

The study protocol was approved by the institutional review board of the Johns Hopkins School of Medicine, MedStar Health Systems, and by the medical director of each dialysis unit. All participants provided informed written consent.

### Data collection

Data were collected by self-report from standardized questionnaires and also during study visits at the Johns Hopkins Institute for Clinical and Translational Research (ICTR). All participants underwent a study visit with detailed cardiovascular evaluations by trained technologists or study staff including echocardiogram, PWV and BP assessments on non-dialysis days to ensure uniformity across the study population. Based on the studies of myocardial stunning during dialysis and at the end of dialysis treatments with many shifts in fluids and electrolytes, the study visit was set to a non-dialysis day in order to standardize the study visits for all dialysis patients and asses their cardiac status at a steady state. Data on all hemodialysis treatment parameters and available laboratory tests during outpatient dialysis therapy were also provided by Davita Clinical Research and MedStar Health Systems.

The echocardiograms (Toshiba Artida, Japan) were performed by three trained technologists at baseline, to determine left ventricular (LV) stroke volume, LV ejection fraction, and LVMI with 4 chamber views and standard calculations for LVM. The M-mode by the parasternal short axis view was used to estimate LVM, as the long axis view can result in the improper alignment and overestimate LV dimensions and mass [17]. All cardiovascular studies were centrally read. Very few participants at baseline had heart failure indicated by low ejection fraction (1 %). Additionally, we estimated pulmonary artery pressures based on measured velocity of the tricuspid regurgitant (TR) jet. Aortic PWV measures were performed supine on the non-fistula arm after at least 5 min of rest using the right carotid and right femoral arteries by four investigators, and the augmentation index was measured using radial tonometry using the Sphygmocor PVx System (AtCor Medical, West Ryde, Australia) device [18]. PWV measurements were performed in the morning under similar conditions in all participants and appropriate quality control measures were performed. Additionally, patients were fasting prior to the assessment [18].

The exposure variables of interest included measures of volume (pulmonary artery pressures and IDWG), and vascular and arterial measurements. Vascular measurements included SBP and diastolic blood pressure (DBP), mean arterial pressure (MAP), pulse pressure, PWV, and augmentation index. Four different measurements of SBP or DBP and pulse pressure at various time points were included. Predialysis BP measures taken at the dialysis units were examined as (1) a three-month average of the predialysis measurements taken using automated BP measurements prior to the study visit and (2) a single measurement closest to the study visit. Non-dialysis BP measures taken at the study vist on a non-dialysis day were examined as: (1) an average of three standardized BP measures conducted using the Omron HEM-907 BP measuring device (Omron Healthcare, Kyoto, Japan) in a seated and (2) an average of three BP measures collected using the same method in a supine position. Volume status was estimated by pulmonary artery pressure based on TR from the echocardiogram [19–21], as the presence of pulmonary hypertension occurs when TR jet velocity exceeds 3.4 m/s [22]. Furthermore, echocardiographic parameters such as left atrial diameters, which may be reflective of the filling pressure and influenced by TR, have been associated with excess volume in hemodialysis (HD) patients [23]. Volume measurement also included IDWG, and this was analyzed as: 1) a three-month average of IDWG measurements taken at the dialysis units, and 2) a measurement closest to the study visit.

The primary endpoint was LVMI calculated by Devereux’s formula [24], as recommended by the American Society of Echocardiography [17], using left ventricular internal diameter (LVID), interventricular septal thickness (IVST) and left ventricular posterior wall thickness (PWT):

$$ \mathrm{LVMI}\left(\mathrm{g}/{\mathrm{m}}^2\right)=\left(0.8\left(1.04\left[{\left(\mathrm{IVST}+\mathrm{LVID}+\mathrm{P}\mathrm{W}\mathrm{T}\right)}^3\hbox{-} {\mathrm{LVID}}^3\right]\right)+0.6\mathrm{g}\right)/\mathrm{B}\mathrm{S}\mathrm{A} $$

LVH was defined as LVMI greater than 116 g/m^2^ in males and LVMI greater than 104 g/m^2^ in females.

Additional variables included self-reported age at enrollment, sex, and ethnicity. Body-mass index (BMI) and waist to hip ratio (WHR) were determined during the ICTR visits. Comorbidities were identified by detailed chart review of medical records and included history of hypertension, hypercholesterolemia, smoking status, diabetes mellitus, coronary artery disease (CAD), congestive heart failure (CHF) and peripheral vascular disease (PVD), dialysis frequency, cause of ESRD, time since first nephrology visit, antihypertensive medications, and the Charlson Comorbidity Index (CCI). Medications were determined at baseline by medical record review from dialysis electronic patient records and participant questionnaire during the study visit. Medications were also brought in for review by the study team. As a result, adherence to medication treatment was not specifically assessed. Antihypertensive medications were grouped into the following categories: (1) Renin-angiotensin-aldosterone system (RAAS) blockade (angiotensin-converting-enzyme inhibitor [ACEi] or angiotensin II receptor blocker [ARB]), (2) β-blocker or α- and β-blocker, (3) α-blocker, vasodilator, or calcium channel blocker, (4) centrally acting agent, and (5) other. Dietary sodium intake was calculated from a 24-h diet recall at the baseline study visit. Dialysis unit hemoglobin data were included as a 3 month average prior to the study visit.

### Statistical analysis

For categorical variables, frequencies and percentages with 95 % confidence intervals (CI) were used; for continuous variables, means with standard deviations (± SD) were used for normally distributed data and median with interquartile range for skewed distributed data.

Multiple analyses were used to examine the association of BP, arterial stiffness, and volume with LVMI. To assess the associations of each vascular, arterial and volume measurement with LVMI, a multivariable linear regression for each vascular, arterial or volume measure was conducted. To assess the independent association of each main explanatory measure with LVMI, all vascular, arterial, and volume measures were simultaneously adjusted for in the final multivariable linear regression model. Each vascular measure was modeled in a separate multivariable model due to the high correlation between the vascular measurements. The multivariable models were constructed using the forward selection model building approach, and included age, sex, ethnicity, BMI, history of hypercholesterolemia, smoking, diabetes mellitus, coronary heart disease, congestive heart failure, cause of end-stage renal disease, time since first nephrology visit, beta blocker and RAAS blockade use, number of antihypertensive medication, and dietary sodium intake. To ensure the consistency and the validity of the final model, all omitted variables were included back into the final model and this indicated that there were no significant changes in the main estimates. Correlation and variance inflation factor tests indicated a low degree of multicollinearity among all factors. Tests of non-linearity indicated that there were no significant non-linear effects. Patients with missing antihypertensive medication data (14 %) were grouped into a separate category. All other missing values for covariates were imputed using the multiple imputation by chained equations method [25]. The imputed variables with missing values were WHR (4.1 %), history of hypercholesterolemia (4.3 %), CCI (2.6 %), dietary sodium intake (5.9 %), and time since first nephrology visit (1.0 %).

Given the high proportion of African American patients in this study, we tested for an interaction between the exposure variables and ethnicity (African Americans vs. non-African Americans) in stratified models. We also stratified the results from the multivariable regression models using pre-identified groups of IDWG (0-2 kg, 2-3 kg, and >3 kg), by β-blocker medication status [26], and by RAAS blockade use.

A two-tailed *P* value of < 0.05 was considered statistically significant. All analyses were performed using Stata/SE 12.0 (StataCorp, College Station, TX).

## Results

Of the 402 participants who completed the study visits with an echocardiogram, 11 were excluded due to poor echocardiographic windows for measurement of LVMI. The final study cohort comprised of 391 patients. Table 1 summarizes the baseline participant, vascular, volume, and echocardiogram measurement characteristics. The study population has mean age at enrollment of 54.7 years, median BMI of 27.9 kg/m^2^ and are predominantly male and African American. Cardiovascular risk factors are common: 57 % with diabetes, 33 % with CAD, 41 % with CHF, 20 % with PVD, and 62 % has a history of smoking. The vast majority is on two to three antihypertensive medications with approximately 62 % taking a beta-blocker and 39 % on any RAAS blockade. A total of 306 patients (78 %) have demonstrable LVH.

**Table 1:** Baseline characteristics of incident hemodialysis participants

Variables	Number of patients	n(%) or mean(±SD) or median(IQR)
	(*N* = 391)	
Age (years)	391	54.7 (±13.2)
Male	391	229 (58.6 %)
Ethnicity		
African American	391	284 (72.6 %)
Non-African American		107 (27.4 %)
Body mass index (kg/m^2^)	391	27.9 (23.8, 33.2)
Waist to hip ratio	375	0.95 (±0.08)
Ever smoker	391	242 (61.9 %)
Hypercholesterolemia	374	225 (57.5 %)
Hypertension	391	390 (99.7 %)
Diabetes mellitus	391	224 (57.3 %)
Coronary artery disease	391	130 (33.3 %)
Congestive heart failure	391	160 (40.9 %)
Peripheral vascular disease	387	77 (19.9 %)
Dietary sodium intake (mg)	368	2219.1 (1370.3, 3045.1)
Hemoglobin (predialysis 3 month average)	361	10.5 (±1.2)
Charlson comorbidity index	381	5 (3, 6)
Medications		
ACEi or ARB	336	154 (46.6 %)
β-blocker or α- and β-blocker	336	242 (61.9 %)
α-blocker or vasoilator or calcium channel blocker	336	271 (69.3 %)
Centrally acting agent	336	47 (12.0 %)
Diuretic	336	80 (20.5 %)
Total number of antihypertensive medications		
1 medication	336	48 (12.3 %)
2 medications		109 (27.9 %)
3 medications		106 (27.1 %)
> 4 medications		73 (18.7 %)
Cause of end-stage renal disease		
Diabetes	391	136 (34.8 %)
Hypertension		100 (25.6 %)
Glomerulonephritis		54 (13.8 %)
Other		62 (15.9 %)
Unknown		39 (10.0 %)
Time since first nephrology visit		
0 months	387	83 (21.2 %)
< 3 months		53 (13.6 %)
3 months - 12 months		68 (17.4 %)
> 12 months		183 (46.8 %)
Three times a week dialysis	386	386 (98.7 %)
Three to four hour dialysis session	389	344 (88.0 %)
Hemodialysis access		
Arteriovenous fistula	389	120 (30.7 %)
Other		271 (69.3 %)
Polyflux dialysis membrane^a^	391	300 (76.7 %)
Vascular Measurements		
Blood pressure (mmHg)		
Predialysis (3 month average)		
Systolic	383	153.0 (±16.8)
Diastolic		84.0 (±11.3)
Predialysis (Prior to study visit)		
Systolic	380	154.9 (±26.0)
Diastolic		84.2 (±15.8)
Non-dialysis (Seated)		
Systolic	387	136.6 (±25.2)
Diastolic		74.5 (±14.9)
Non-dialysis (Supine)		
Systolic	367	148.0 (±25.4)
Diastolic		79.1 (±15.2)
Mean arterial pressure (Non-dialysis)	370	103.0 (±19.7)
Pulse pressure		
Predialysis (3 months average)	383	69.0 (±13.1)
Predialysis (Prior to study visit)	380	69.7 (±20.1)
Non-dialysis (Seated)	387	62.1 (±17.9)
Non-dialysis (Supine)	367	68.8 (±18.3)
Arterial Stiffness Measurements		
Pulse wave velocity (m/s)	337	11.2 (±3.9)
Central augmentation index (%)	365	25.4 (19.0, 34.0)
Volume Measurements		
Tricuspid regurgitation (Non-dialysis)	211	30.3 (±10.8)
Intradialytic weight gain (3 month average) (kg)	383	2.3 (±1.1)
Intradialytic weight gain (Prior to study visit) (kg)	380	2.6 (±1.9)
Echocardiogram Measurements		
Left ventricular ejection fraction	391	65.3 (±12.0)
Left ventricular mass (g)	391	286.5 (±98.7)
Left ventricular mass index (g/m^2^)	391	150.0 (±49.0)
Left ventricular hypertrophy^b^	391	306 (78.3 %)

*ARB* angiotensin II receptor blocker, *ACEi* angiotensin-converting-enzyme inhibitor

^a^Polyflux, Gambro dialyzer with a polymer blend of polyarylethersulfone, polyvinylpyrrolidone, and polyamide dialysis membrane

^b^LVMI ≥ 116 g/m^2^ in males, ≥ 104 g/m^2^ in females

**Fig. 1:**
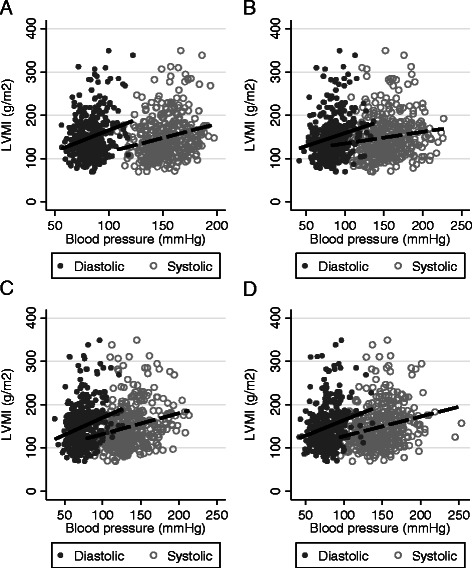
Scatterplots of left ventricular mass index (LVMI) and systolic and diastolic blood pressure at various time points as **a** three-month average of the predialysis measurement prior to the study visit; **b** predialysis measurement prior to the study visit; **c** seated non-dialysis BP measurement and **d** supine non-dialysis measurement

**Fig. 2:**
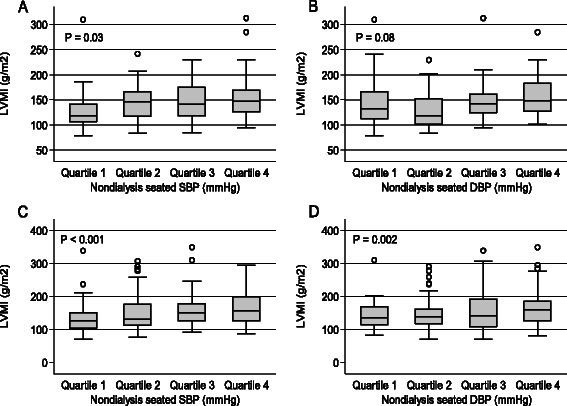
Boxplot of left ventricular mass index (LVMI) and quartiles of systolic or diastolic blood pressures measured as a seated non-dialysis BP measurement stratified by ethnicity **a** and **b** non-African Americans and **c** and **d** African American participants

Figure 1 illustrates side-by-side scatterplots of LVMI, SBP, and DBP by type of BP measurement. In all four measurements of BP, SBP and DBP have graded linear associations with LVMI; however, DBP consistently shows a steeper slope with LVMI than SBP. These patterns are consistent regardless of timing of the BP measurement. Figure 2 illustrates the quartiles of non-dialysis seated BP measurements and trends of LVMI, stratified by ethnicity. The plots demonstrate a significant positive association between quartiles of SBP and DBP, and LVMI among African Americans. Among non-African Americans, only SBP is significantly associated with LVMI. Similar associations are depicted using other BP measurements (Additional file 1: Figure S1–S3).

The associations of vascular and volume measures with LVMI are examined and shown in Table 2. The unadjusted linear regression model demonstrates that, among vascular measures, BP, pulse pressure, and MAP are all associated with higher LVMI. In particular, all SBPs and DBPs, regardless of the timing of the measurements, are most significantly associated with LVMI. Among volume measures, only IDWG just prior to the study visit is significantly associated with higher LVMI. Adjusting for baseline covariates demonstrates that BP measures remains significantly associated with higher LVMI and the association with IDWG is attenuated. In particular, DBPs are the most important factors among BPs. Measures of arterial stiffness or pulmonary pressure, after multivariable adjustment, are not associated with LVMI.

**Table 2:** Association of vascular, arterial, and volume measures with LVMI by linear regression among incident hemodialysis participants

Variables	n	Univariable		Multivariable^a^	
		β (95 % CI)	*P*	β (95 % CI)	*P*
Vascular measurements					
Blood pressure (per 10 mmHg)					
Predialysis (3 month average)	383				
Systolic		6.51 (3.69, 9.33)	<0.001	7.26 (4.30, 10.23)	<0.001
Diastolic		9.71 (5.52, 13.90)	<0.001	10.05 (5.06, 15.04)	<0.001
Predialysis (Prior to study visit)	380				
Systolic		2.69 (0.83, 4.56)	0.005	2.46 (0.57, 4.35)	0.01
Diastolic		5.95 (2.92, 8.99)	<0.001	3.93 (0.67, 7.20)	0.02
Non-dialysis (Seated)	387				
Systolic		4.83 (2.94, 6.72)	<0.001	5.00 (3.11, 6.89)	<0.001
Diastolic		7.87 (4.68, 11.06)	<0.001	7.28 (3.79, 10.77)	<0.001
Non-dialysis (Supine)	367				
Systolic		4.54 (2.62, 6.46)	<0.001	4.72 (2.72, 6.72)	<0.001
Diastolic		7.13 (3.91, 10.35)	<0.001	6.25 (2.64, 9.85)	0.001
Mean arterial pressure (Non-dialysis)	370	0.57 (0.33, 0.82)	<0.001	0.56 (0.29, 0.82)	<0.001
Pulse pressure					
Predialysis (3 months average)	383	0.35 (−0.02, 0.72)	0.07	0.71 (0.29, 1.13)	0.001
Predialysis (Prior to study visit)	380	0.12 (−0.13, 0.37)	0.34	0.26 (−0.01, 0.53)	0.05
Non-dialysis (Seated)	387	0.41 (0.13, 0.68)	0.003	0.65 (0.36, 0.94)	<0.001
Non-dialysis (Supine)	367	0.38 (0.11, 0.65)	0.006	0.60 (0.30, 0.89)	<0.001
Arterial stiffness measurements					
Pulse wave velocity (m/s)	337	−0.42 (−1.78, 0.94)	0.55	0.19 (−1.41, 1.79)	0.82
Central augmentation index	365	−0.12 (−0.48, 0.25)	0.53	−0.03 (−0.41, 0.36)	0.88
Volume measurements					
Tricuspid regurgitation (Non-dialysis)	211	0.32 (−0.29, 0.92)	0.30	0.10 (−0.51, 0.70)	0.76
Intradialytic weight gain (3 month average)	383	3.75 (−0.51, 8.01)	0.09	4.36 (−0.26, 8.98)	0.06
Intradialytic weight gain (Prior to study visit)	380	3.77 (1.17, 6.37)	0.005	3.25 (0.67, 5.83)	0.01

^a^Adjusted model included age, sex, ethnicity, body mass index, history of hypercholesterolemia, smoking, diabetes mellitus, coronary heart disease, congestive heart failure, cause of end-stage renal disease, time since first nephrology visit, beta blocker use, RAAS blockade use, number of antihypertensive medication, and dietary sodium intake

Table 3 demonstrates the independent associations of non-dialysis seated BP, PWV, and volume measures with LVMI using a multivariable linear regression model that simultaneously adjusted for the main explanatory measures and other covariates. Among all BP, arterial, and volume measurements, SBP and DBP are independently associated with LVMI. Similar associations are demonstrated using other vascular measures (predialysis, pulse pressure, mean arterial pressure; Additional file 1: Table S1–S4). PWV and IDWG measures are not independently associated with LVMI.

**Table 3:** Independent associations of non-dialysis seated blood pressure, arterial, and volume measures with LVMI by linear regression among incident hemodialysis participants

Variables	Model 1^a^		Model 2^b^	
	(*n* = 177)		(*n* = 177)	
	β (95 % CI)	*P*	β (95 % CI)	*P*
Vascular measurements				
Blood pressure (per 10 mmHg)				
Non-dialysis seated systolic	5.64 (2.78, 8.49)	<0.001	-	-
Non-dialysis seated diastolic	-	-	7.29 (2.26, 12.31)	0.005
Arterial stiffness measurement				
Pulse wave velocity (m/s)	−0.05 (−2.44, 2.34)	0.97	0.41 (−2.01, 2.84)	0.74
Central augmentation index	−0.36 (−0.88, 0.17)	0.19	−0.38 (−0.93, 0.16)	0.17
Volume measurement				
Tricuspid regurgitation (Non-dialysis)	−0.11 (−0.76, 0.54)	0.74	−0.04 (−0.70, 0.62)	0.90
Intradialytic weight gain (3-month average)	5.84 (−1.51, 13.19)	0.12	7.67 (0.06, 15.28)	0.05
Intradialytic weight gain (Prior to study visit)	2.03 (−1.91, 5.97)	0.31	1.69 (−2.33, 5.71)	0.41

^a^Model 1 included non-dialysis seated systolic blood pressure, pulse wave velocity, central augmentation index, tricuspid regurgitation, intradialytic weight gain (3-month average), and intradialytic weight gain (prior to study visit), as well as baseline age, sex, ethnicity, body mass index, history of hypercholesterolemia, smoking, diabetes mellitus, coronary heart disease, congestive heart failure, cause of end-stage renal disease, time since first nephrology visit, beta blocker use, RAAS blockade use, number of antihypertensive medication, and dietary sodium intake

^b^Model 2 included non-dialysis seated diastolic blood pressure, pulse wave velocity, central augmentation index, tricuspid regurgitation, intradialytic weight gain (3-month average), and intradialytic weight gain (prior to study visit), as well as baseline age, sex, ethnicity, body mass index, history of hypercholesterolemia, smoking, diabetes mellitus, coronary heart disease, congestive heart failure, cause of end-stage renal disease, time since first nephrology visit, beta blocker use, RAAS blockade use, number of antihypertensive medication, and dietary sodium intake

Given the high proportion of African American patients, we first examined the interaction between ethnicity and the exposure variables, and then stratified the results from the adjusted model (Additional file 1: Table S5). The stratified analysis by ethnicity demonstrates that in African Americans, most BPs, pulse pressures, and MAP are significantly associated with LVMI, whereas in non-African Americans, few factors were associated with LVMI (Additional file 1: Table S5). There are no significant interactions between ethnicity and the explanatory variables.

We also postulated that the associations of BPs and LVMI might not be constant across participants with varying IDWG. In the stratified analyses (Additional file 1: Table S6), among those in the lowest IDWG group (<2 kg), most BP measurements and pulse pressures are significantly associated with LVMI. In the intermediate IDWG group (2–3 kg), most BP measures are significantly associated with LVMI. In the highest IDWG group (>3 kg), no measures are associated with LVMI; however, the sample size is extremely small.

Based on recent trial findings that demonstrate a difference in cardiovascular morbidity between β-blocker- and ACEi-based therapy [26], we also stratified by β-blocker medication status (Additional file 1: Table S7) and RAAS blockade use (Additional file 1: Table S8). Among patients who received no β-blockers, some BP measures and IDWG prior to study visit are significantly associated with higher LVMI. Among patients who received β-blocker medications, most vascular measures are associated with higher LVMI. There are no significant differences in the mean BPs between patients who received β-blockers and did not receive β-blockers. In patients who received RAAS blockade, most vascular measures are significantly associated with higher LVMI. In patients who did not receive RAAS blockade, few measures are associated with higher LVMI. There are also no differences in the mean BPs between patients who did and did not receive RAAS blockade.

## Discussion

Among an ethnically diverse cohort incident to hemodialysis, measures of afterload are consistently and independently associated with higher LVMI in a linear graded response even after controlling for other cardiovascular disease risk factors and antihypertensive medications. Arterial stiffness and pulmonary pressure are not associated with LVMI. This suggests that, of all vascular, arterial, and volume measures, afterload reduction as measured by BP is an important factor associated with higher LVMI in those starting dialysis, and thus significant in managing cardiovascular risk for those patients during the early initiation of hemodialysis.

Our study extends findings from the longitudinal Chronic Renal Insufficiency Cohort (CRIC) study, which examines changes in LV structure and function in patients transitioning from CKD to ESRD but could not examine the role of clinical factors associated with LV disease [27]. We are able to demonstrate, in a similar but larger population of incident HD patients, significant associations of modifiable risk factors, in particular, blood and pulse pressures, with LVM. Our findings are also consistent in a subgroup of patients without a prior history of CHF (Additional file 1: Table S9). We report fewer patients with LVH and mostly with preserved cardiac function, which may be reflective of a younger and more ethnically diverse study population. Our findings also confirm and extend findings from the Erythropoietin Normalization trial that showed a significant association between higher SBP and LVM [5], as we are able to demonstrate consistently higher LVMI with either SBPs or DBPs at various time points and controlling for measures of volume status and arterial stiffness and not just SBP. Furthermore, no significant associations between hemoglobin and LVMI were found in our analysis (Additional file 1: Table S10). Recent reviews have suggested using non-dialysis BPs for management, however, it is complicated to obtain morning BPs unless patients are capable of home monitoring [28, 29]. A 24-h ambulatory BP monitoring would provide the best BP measurements [30], but this is costly and impractical for all dialysis patients [31]. Although both systolic and diastolic BPs are associated with higher LVMI in this study, DBP consistently shows a stronger association with LVMI in comparison to SBP. This finding deviates from earlier studies where higher predialysis SBP is the main arterial hemodynamic factor associated with LVH progression [32]. The stronger association of DBP with LVMI in our study could be attributed to the predominantly African American study population or due to a severely affected diseased population. Comparison of SBP, DBP and pulse pressure with mortality on dialysis patients demonstrated that DBP significantly attenuates the association of SPB and pulse pressure on mortality and thus is an important factor leading to mortality [33]. The association between higher DBP and higher LV mass may be indicative of lower LV diastolic function in our cohort, which is often a consequence of LVH. A meta-analysis of trials has also demonstrated that the reduction of diastolic BP in particular is important in the regression of LVH [34]. Nonetheless, the direction of effect is consistent for either systolic or diastolic pressures.

Despite the findings of previous studies, evidence to guide BP management in dialysis patients still remains sparse [30]. The guidelines from the Kidney Disease Outcomes Quality Initiative and the Joint National Committee suggest that the goal BP should be less than 140/90 mmHg [35, 36], however, the Kidney Disease: Improving Global Outcomes states that these recommendations are largely based on expert opinion and weak evidence, and does not provide a recommendation regarding BP management in dialysis patients [30]. Our results show that approximately 79 % of those receiving dialysis with a predialysis BP greater than 140/90 mmHg had higher LVMI. Furthermore, our study reflects the proportion of patients at dialysis initiation as they transition from advanced CKD to hemodialysis, and this provides a window to potentially mitigate cardiovascular risk on dialysis by aggressively treating high BP at early stages of dialysis. We are also able to demonstrate, in our large incident cohort, that BP is associated with higher LVMI regardless of timing. Clinicians could use predialysis measures for adjustment in medical management.

Other controversies regarding the BP management in dialysis patients include the effect of BP-lowering medications, such as RAAS inhibitors, β-blockers, and calcium channel blockers, on the reduction of LVMI in hypertensive patients [37, 38]. The Hypertension in Hemodialysis Patients Treated with Atenolol or Lisinopril (HDPAL) trial demonstrates the beneficial effect of β-blocker to RAAS blockade in preventing cardiovascular morbidity [26]. Our findings suggest that those on either a β-blocker or RAAS blockade may have a protective role but this needs to be confirmed by larger randomized controlled trials with specifically targeted BP levels.

Contrary to past studies, we do not find that vascular stiffness was significantly associated with LVMI [12]. Arterial stiffness may be a factor associated with LV disease over prolonged dialysis, as vascular calcification can worsens and contribute to increased arterial stiffness [12, 39]. Our study also includes incident and significantly younger HD patients, whereas most studies include prevalent and older patients over age 65 years, which accounts for a higher burden of arterial stiffness due to prolonged dialysis as well as the natural process of aging. Studies with prevalent patients also introduce a survivor bias, which needs to be considered when determining risk factors predicting cardiovascular disease. Though we did not observe a direct association between pulse wave velocity and LVMI, the association between higher pulse pressure, which is determined by arterial compliance and the intensity of wave reflections that are influenced by arterial stiffness, and higher LVMI suggests that reduced arterial compliance and distensibility may still have an underlying role and potentially contribute to LVH [40].

Lastly, IDWG is associated with LVMI, however, the association is attenuated after adjustment for other confounders. Another surrogate measure of volume status, pulmonary pressure, is not significantly associated with higher LVMI. In our study population of younger and diverse ethnicities, adjusting for dietary sodium intake does not modify any associations. The results of our study likely differ based on the change in residual kidney function of dialysis patients over years. Most of our cohort is studied within the first 6 months from diagnosis. This suggests that during the transition from reaching ESRD to receiving dialysis, IDWG management may not be a significant contributor until there is loss of residual function.

A few limitations of this study include the cross-sectional study design, which does not provide evidence of a temporal relationship and would require a trial to demonstrate normalization of blood pressure. Other measures of volume status and pulmonary pressure and preload should also be considered in future studies, as tricuspid regurgitation by echocardiography alone may not be as accurate as newer methods such as ultrasounds to detect lung congestion [41], and TR velocity does not clearly reflect LV preload in the presence of pulmonary vascular disease even though few had documented disease. Nonetheless, pulmonary hypertension is often observed together with volume overload, and has been associated with fluid volume status in past studies that showed a strong association between left atrial diameter and chronic volume overload [42]. Studies have also shown that after a series of ultrafiltration sessions, thereby treating volume overload, tricuspid regurgitation disappeared in most patients [43]. Furthermore, it is difficult to accurately measure volume status using one type of measurement; therefore we used additional factors such as IDWG, which is clinically relevant and available, to help estimate volume status.

Despite the limitations, this well characterized cohort of incident hemodialysis patients has been assessed in a consistent manner on non-dialysis days with standardized questions, adjudicated comorbidities and methods for cardiovascular assessment, as well as extensive dialysis unit BP and IDWG data. Moreover, our study cohort is predominantly African American (72.6 %), often underrepresented in many studies or trials, despite comprising 10.4 % of the Medicare end stage renal disease population. Understanding cardiovascular risk in this high risk population is important [44].

## Conclusion

Among a younger and incident hemodialysis population, afterload as measured by BP using either predialysis or interdialytic measures, is most associated with higher LVMI. This suggests that future studies should consider the use of various BP measures in examining the impact of BP on LVM and cardiovascular disease. Findings from such studies could suggest that high BP should be more aggressively treated to promote LVH regression in incident hemodialysis patients.

## Additional file

Additional file 1: Figure S1. Boxplot of left ventricular mass index (LVMI) and quartiles of systolic or diastolic blood pressures measured as predialysis 3-month average BP measurement stratified by ethnicity. **Figure S2:** Boxplot of left ventricular mass index (LVMI) and quartiles of systolic or diastolic blood pressures measured as prior to clinic BP measurement stratified by ethnicity. **Figure S3:** Boxplot of left ventricular mass index (LVMI) and quartiles of systolic or diastolic blood pressures measured as non-dialysis supine BP measurement stratified by ethnicity. **Table S1:** Independent associations of predialysis blood pressure, arterial, and volume measures with LVMI by linear regression among incident hemodialysis participants. **Table S2:** Independent associations of blood pressure prior to study visit, arterial, and volume measures withLVMI by linear regression among incident hemodialysis participants. **Table S3:** Independent associations of mean arterial pressure, arterial, and volume measures with LVMI by linear regression among incident hemodialysis participants. **Table S4:** Independent associations of pulse pressures, arterial, and volume measures with LVMI by linear regression among incident hemodialysis participants. **Table S5:** Association of preload and afterload measures with LVMI by linear regression among incident hemodialysis participants stratified by ethnicity. **Table S6:** Association of preload and afterload measures with LVMI by linear regression among incidenthemodialysis participants stratified by 3 month average IDWG groups. **Table S7:** Association of preload and afterload measures with LVMI by linear regression among incident hemodialysis participants stratified by β-blocker medication. **Table S8:** Association of preload and afterload measures with LVMI by linear regression among incident hemodialysis participants stratified by renin-angiotensin-aldosterone system (RAAS) blockade use. **Table S9:** Association of preload and afterload measures with LVMI by linear regression among incident hemodialysis participants stratified by history of congestive heart failure. **Table S10:** Association of hemoglobin with LVMI by linear regression among incident hemodialysis participants. (DOCX 97 kb)

## Abbreviations

ACEi: Angiotensin converting enzyme inhibitor
ARB: Angiotensin-II receptor blocker
BMI: Body-mass index
BP (SBP: DBP), Blood pressure (systolic blood pressure, diastolic blood pressure)
CAD: Coronary artery disease
CCI: Charlson comorbidity index
CHF: Congestive heart failure
CI: Confidence interval
CKD: Chronic kidney disease
ESRD: End-stage renal disease
HD: Hemodialysis
ICTR: Institute for Clinical and Translational Research
IDWG: Intradialytic weight gain
IVST: Interventricular septal thickness
LV: Left ventricle
LVH: Left ventricular hypertrophy
LVID: Left ventricular internal diameter
LVM: Left ventricular mass
LVMI: Left ventricular mass index
MAP: Mean arterial pressure
PACE study: Predictors of Arrhythmic and Cardiovascular Risk in End-Stage Renal Disease study
PVD: Peripheral vascular disease
PWT: Left ventricular wall thickness
PWV: Pulse wave velocity
RAAS: Renin-angiotensin-aldosterone system
SD: Standard deviation
TR: Tricuspid regurgitation
WHR: Waist to hip ratio
