# Supplementary material for: Cross-sectional association of volume, blood pressures, and aortic stiffness with left ventricular mass in incident hemodialysis patients: the Predictors of Arrhythmic and Cardiovascular Risk in End-Stage Renal Disease (PACE) study
Source: BMC Nephrol. 2015 Aug 7;16:131. doi: 10.1186/s12882-015-0131-4 (PMC4528691; doi:10.1186/s12882-015-0131-4)
Supplement: Additional file 1: Figure S1. — Boxplot of left ventricular mass index (LVMI) and quartiles of systolic or diastolic blood pressures measured as predialysis 3-month average BP measurement stratified by ethnicity. Figure S2: Boxplot of left ventricular mass index (LVMI) and quartiles of systolic or diastolic blood pressures measured as prior to clinic BP measurement stratified by ethnicity. Figure S3: Boxplot of left ventricular mass index (LVMI) and quartiles of systolic or diastolic blood pressures measured as non-dialysis supine BP measurement stratified by ethnicity. Table S1: Independent associations of predialysis blood pressure, arterial, and volume measures with LVMI by linear regression among incident hemodialysis participants. Table S2: Independent associations of blood pressure prior to study visit, arterial, and volume measures withLVMI by linear regression among incident hemodialysis participants. Table S3: Independent associations of mean arterial pressure, arterial, and volume measures with LVMI by linear regression among incident hemodialysis participants. Table S4: Independent associations of pulse pressures, arterial, and volume measures with LVMI by linear regression among incident hemodialysis participants. Table S5: Association of preload and afterload measures with LVMI by linear regression among incident hemodialysis participants stratified by ethnicity. Table S6: Association of preload and afterload measures with LVMI by linear regression among incidenthemodialysis participants stratified by 3 month average IDWG groups. Table S7: Association of preload and afterload measures with LVMI by linear regression among incident hemodialysis participants stratified by β-blocker medication. Table S8: Association of preload and afterload measures with LVMI by linear regression among incident hemodialysis participants stratified by renin-angiotensin-aldosterone system (RAAS) blockade use. Table S9: Association of preload and afterload measures with LVMI by linear regression among incide [file 12882_2015_131_MOESM1_ESM.docx]

**Supplementary Figure 1.** Boxplot of left ventricular mass index (LVMI) and quartiles of systolic or diastolic blood pressures* measured as predialysis 3-month average BP measurement stratified by ethnicity**

* SBP Quartile 1: 107.9 - 140.8 mmHg, Quartile 2: 140.9 – 152.9 mmHg, Quartile 3: 153.1 – 164.5 mmHg, Quartile 4: 164.6 – 197.5 mmHg; DBP Quartile 1: 56.4 – 75.6 mmHg, Quartile 2: 75.8 – 83.8 mmHg, Quartile 3: 83.9 – 90.9 mmHg, Quartile 4: 91.0 – 122.3 mmHg

** A) and B) non-African American and C) and D) African American participants

**Supplementary Figure 2.** Boxplot of left ventricular mass index (LVMI) and quartiles of systolic or diastolic blood pressures* measured as prior to clinic BP measurement stratified by ethnicity**

* SBP Quartile 1: 82 - 136 mmHg, Quartile 2: 137 - 153 mmHg, Quartile 3: 153 - 171 mmHg, Quartile 4: 171 - 227 mmHg; DBP Quartile 1: 41 - 74 mmHg, Quartile 2: 74 - 83 mmHg, Quartile 3: 83 – 93 mmHg, Quartile 4: 94 - 137 mmHg

** A) and B) non-African Americans and C) and D) African American participants

**Supplementary Figure 3.** Boxplot of left ventricular mass index (LVMI) and quartiles of systolic or diastolic blood pressures* measured as non-dialysis supine BP measurement stratified by ethnicity**

* SBP Quartile 1: 94 – 129.5 mmHg, Quartile 2: 129.5 – 145.5 mmHg, Quartile 3: 145.5 - 163 mmHg, Quartile 4: 163.5 – 253.5 mmHg; DBP Quartile 1: 42 – 68.5 mmHg, Quartile 2: 69 – 77.5 mmHg, Quartile 3: 77.5 – 88.5 mmHg, Quartile 4: 89 – 137.5 mmHg

** A) and B) non-African Americans and C) and D) African American participants

**Supplementary Table 1.** Independent associations of predialysis blood pressure, arterial, and volume measures with LVMI by linear regression among incident hemodialysis participants

| **Variables** | **Model 1* (n = 177)** | | **Model 2** (n = 177)** | |
| --- | --- | --- | --- | --- |
|  | **β (95% CI)** | ***P*** | **β (95% CI)** | ***P*** |
|  |  |  |  |  |
| **Vascular measurements** |  |  |  |  |
| **Blood pressure (per 10 mmHg)** |  |  |  |  |
| Predialysis systolic | 5.80 (0.89, 10.72) | 0.02 |  |  |
| Predialysis diastolic |  |  | 5.03 (-2.8, 12.86) | 0.20 |
|  |  |  |  |  |
| **Arterial stiffness measurement** |  |  |  |  |
| **Pulse wave velocity (m/s)** | -0.12 (-2.64, 2.39) | 0.92 | 0.32 (-2.19, 2.83) | 0.80 |
| **Central augmentation index** | -0.25 (-0.79, 0.30) | 0.37 | -0.30 (-0.85, 0.26) | 0.29 |
|  |  |  |  |  |
| **Volume measurement** |  |  |  |  |
| **Tricuspid regurgitation (Non-dialysis)** | 0.01 (-0.65, 0.68) | 0.96 | 0.09 (-0.58, 0.76) | 0.79 |
| **Intradialytic weight gain (3-month average)** | 5.01 (-2.61, 12.64) | 0.19 | 5.91 (-1.77, 13.59) | 0.13 |
| **Intradialytic weight gain (Prior to study visit)** | 2.00 (-2.08, 6.08) | 0.33 | 1.47 (-2.63, 5.58) | 0.47 |
| ***** Model 1 included predialysis systolic blood pressure, pulse wave velocity, central augmentation index, tricuspid regurgitation, intradialytic weight gain (3-month average), and intradialytic weight gain (prior to study visit), as well as baseline age, sex, ethnicity, body mass index, history of hypercholesterolemia, smoking, diabetes mellitus, coronary heart disease, congestive heart failure, cause of end-stage renal disease, time since first nephrology visit, beta blocker use, RAAS blockade use, number of antihypertensive medication, and dietary sodium intake  ****** Model 2 included predialysis diastolic blood pressure, pulse wave velocity, central augmentation index, tricuspid regurgitation, intradialytic weight gain (3-month average), and intradialytic weight gain (prior to study visit), as well as baseline age, sex, ethnicity, body mass index, history of hypercholesterolemia, smoking, diabetes mellitus, coronary heart disease, congestive heart failure, cause of end-stage renal disease, time since first nephrology visit, beta blocker use, RAAS blockade use, number of antihypertensive medication, and dietary sodium intake | | | | |

**Supplementary Table 2.** Independent associations of blood pressure prior to study visit, arterial, and volume measures with LVMI by linear regression among incident hemodialysis participants

| **Variables** | **Model 1* (n = 177)** | | **Model 2** (n = 177)** | |
| --- | --- | --- | --- | --- |
|  | **β (95% CI)** | ***P*** | **β (95% CI)** | ***P*** |
|  |  |  |  |  |
| **Vascular measurements** |  |  |  |  |
| **Blood pressure (per 10 mmHg)** |  |  |  |  |
| Prior to study visit (systolic) | 3.43 (0.74, 6.13) | 0.01 |  |  |
| Prior to study visit (diastolic) |  |  | 6.57 (2.19, 10.95) | 0.004 |
|  |  |  |  |  |
| **Arterial stiffness measurement** |  |  |  |  |
| **Pulse wave velocity (m/s)** | 0.27 (-2.18, 2.72) | 0.82 | 0.27 (-2.15, 2.70) | 0.82 |
| **Central augmentation index** | -0.24 (-0.78, 0.30) | 0.38 | -0.31 (-0.85, 0.23) | 0.25 |
|  |  |  |  |  |
| **Volume measurement** |  |  |  |  |
| **Tricuspid regurgitation (Non-dialysis)** | 0.02 (-0.64, 0.68) | 0.95 | -0.31 (-0.85, 0.23) | 0.25 |
| **Intradialytic weight gain (3-month average)** | 6.31 (-1.26, 13.88) | 0.10 | 0.03 (-0.63, 0.68) | 0.94 |
| **Intradialytic weight gain (Prior to study visit)** | 1.27 (-2.77, 5.32) | 0.53 | 7.00 (-0.54, 14.53) | 0.06 |
| ***** Model 1 included systolic blood pressure prior to study visit, pulse wave velocity, central augmentation index, tricuspid regurgitation, intradialytic weight gain (3-month average), and intradialytic weight gain (prior to study visit), as well as baseline age, sex, ethnicity, body mass index, history of hypercholesterolemia, smoking, diabetes mellitus, coronary heart disease, congestive heart failure, cause of end-stage renal disease, time since first nephrology visit, beta blocker use, RAAS blockade use, number of antihypertensive medication, and dietary sodium intake  ****** Model 2 included diastolic blood pressure prior to study visit, pulse wave velocity, central augmentation index, tricuspid regurgitation, intradialytic weight gain (3-month average), and intradialytic weight gain (prior to study visit), as well as baseline age, sex, ethnicity, body mass index, history of hypercholesterolemia, smoking, diabetes mellitus, coronary heart disease, congestive heart failure, cause of end-stage renal disease, time since first nephrology visit, beta blocker use, RAAS blockade use, number of antihypertensive medication, and dietary sodium intake | | | | |

**Supplementary Table 3.** Independent associations of mean arterial pressure, arterial, and volume measures with LVMI by linear regression among incident hemodialysis participants

| **Variables** | **Model 1* (n = 176)** | |
| --- | --- | --- |
|  | **β (95% CI)** | ***P*** |
|  |  |  |
| **Vascular measurements** |  |  |
| **Mean arterial pressure** | 0.77 (0.35, 1.19) | < 0.001 |
|  |  |  |
| **Arterial stiffness measurement** |  |  |
| **Pulse wave velocity (m/s)** | -0.72 (-3.21, 1.76) | 0.56 |
| **Central augmentation index** | -0.70 (-1.28, -0.13) | 0.02 |
|  |  |  |
| **Volume measurement** |  |  |
| **Tricuspid regurgitation (Non-dialysis)** | -0.03 (-0.68, 0.61) | 0.92 |
| **Intradialytic weight gain (3-month average)** | 6.43 (-0.93, 13.80) | 0.08 |
| **Intradialytic weight gain (Prior to study visit)** | 1.60 (-2.39, 5.59) | 0.42 |
| ***** Model 1 included mean arterial pressure, pulse wave velocity, central augmentation index, tricuspid regurgitation, intradialytic weight gain (3-month average), and intradialytic weight gain (prior to study visit), as well as baseline age, sex, ethnicity, body mass index, history of hypercholesterolemia, smoking, diabetes mellitus, coronary heart disease, congestive heart failure, cause of end-stage renal disease, time since first nephrology visit, beta blocker use, RAAS blockade use, number of antihypertensive medication, and dietary sodium intake | | |

**Supplementary Table 4.** Independent associations of pulse pressures, arterial, and volume measures with LVMI by linear regression among incident hemodialysis participants

| **Variables** | **Model 1* (n = 177)** | **Model 2* (n = 177)** | | | **Model 3* (n = 177)** | **Model 4* (n = 175)** | |  |
| --- | --- | --- | --- | --- | --- | --- | --- | --- |
|  | **β (95% CI)** | | **β (95% CI)** | **β (95% CI)** | | | **β (95% CI)** | |
|  |  |  |  |  |  |  |  |  |
| **Vascular measurements** |  | |  |  | | |  | |
| **Pulse pressure** |  | |  |  | | |  | |
| Predialysis | 0.76 (0.06, 1.45)^†^ | |  |  | | |  | |
| Prior to study visit |  | | 0.23 (-0.19, 0.64)^†^ |  | | |  | |
| Non-dialysis (seated) |  | |  | 0.79 (0.34, 1.25)^†^ | | |  | |
| Non-dialysis (supine) |  | |  |  | | | 0.61 (0.14, 1.09)^†^ | |
|  |  | |  |  | | |  | |
| **Arterial stiffness measurement** |  | |  |  | | |  | |
| **Pulse wave velocity (m/s)** | 0.05 (-2.44, 2.55) | | 0.48 (-2.01, 2.97) | -0.13 (-2.56, 2.3) | | | -0.33 (-2.88, 2.21) | |
| **Central augmentation index** | -0.19 (-0.73, 0.36) | | -0.23 (-0.78, 0.33) | -0.26 (-0.79, 0.27) | | | -0.37 (-0.93, 0.18) | |
|  |  | |  |  | | |  | |
| **Volume measurement** |  | |  |  | | |  | |
| **Tricuspid regurgitation** | 0.03 (-0.64, 0.7) | | 0.09 (-0.59, 0.76) | -0.03 (-0.68, 0.63) | | | -0.06 (-0.72, 0.61) | |
| **IDWG (3 month average)** | 4.79 (-2.89, 12.47) | | 5.82 (-1.88, 13.52) | 3.93 (-3.59, 11.46) | | | 5.01 (-2.63, 12.64) | |
| **IDWG (Prior to study visit)** | 2.08 (-2.03, 6.2) | | 1.36 (-2.75, 5.48) | 1.98 (-2, 5.97) | | | 1.64 (-2.48, 5.76) | |
| ^†^ p < 0.05  ***** Model includes predialysis pulse pressure (Model 1) or prior to study visit pulse pressure (Model 2) or non-dialysis seated pulse pressure (Model 3) or non-dialysis supine pulse pressure (Model 4), and pulse wave velocity, central augmentation index, tricuspid regurgitation, intradialytic weight gain (3-month average), and intradialytic weight gain (prior to study visit), baseline age, sex, ethnicity, body mass index, history of hypercholesterolemia, smoking, diabetes mellitus, coronary heart disease, congestive heart failure, cause of end-stage renal disease, time since first nephrology visit, beta blocker use, RAAS blockade use, number of antihypertensive medication, and dietary sodium intake | | | | | | | |  |

**Supplementary Table 5.** Association of preload and afterload measures with LVMI by linear regression among incident hemodialysis participants stratified by ethnicity

|  |  |  |  |  |  |  |  |  |
| --- | --- | --- | --- | --- | --- | --- | --- | --- |
| **Variables** | **Adjusted* (Non African American)** | | |  | **Adjusted* (African American)** | | |  |
|  | **n** | **β (95% CI)** | ***P*** |  | **n** | **β (95% CI)** | ***P*** |  |
|  |  |  |  |  |  |  |  |  |
| **Vascular measurements** |  |  |  |  |  |  |  |  |
| **Blood pressure (per 10 mmHg)** |  |  |  |  |  |  |  |  |
| **Predialysis (3 month average)** | 106 |  |  |  | 277 |  |  |  |
| Systolic |  | 5.80 (0.46, 11.13) | 0.03 |  |  | 8.33 (4.51, 12.16) | < 0.001 |  |
| Diastolic |  | 6.04 (-3.50, 15.58) | 0.21 |  |  | 12.61 (6.47, 18.76) | < 0.001 |  |
| **Predialysis (Prior to study visit)** | 105 |  |  |  | 275 |  |  |  |
| Systolic |  | 3.04 (-0.21, 6.30) | 0.07 |  |  | 2.10 (-0.31, 4.51) | 0.09 |  |
| Diastolic |  | 0.93 (-5.54, 7.40) | 0.78 |  |  | 4.82 (0.82, 8.81) | 0.02 |  |
| **Non-dialysis (Seated)** | 106 |  |  |  | 281 |  |  |  |
| Systolic |  | 4.16 (0.51, 7.81) | 0.03 |  |  | 5.28 (2.97, 7.60) | < 0.001 |  |
| Diastolic |  | 4.51 (-2.42, 11.43) | 0.20 |  |  | 7.86 (3.57, 12.16) | < 0.001 |  |
| **Non-dialysis (Supine)** | 103 |  |  |  | 264 |  |  |  |
| Systolic |  | 2.15 (-1.40, 5.71) | 0.23 |  |  | 6.02 (3.55, 8.49) | < 0.001 |  |
| Diastolic |  | 1.27 (-5.91, 8.45) | 0.73 |  |  | 8.48 (4.05, 12.91) | < 0.001 |  |
| **Mean arterial pressure (Non-dialysis)** | 102 | 0.21 (-0.29, 0.70) | 0.41 |  | 268 | 0.74 (0.42, 1.06) | < 0.001 |  |
| **Pulse pressure** |  |  |  |  |  |  |  |  |
| Predialysis (3 months average) | 106 | 0.78 (0.02, 1.54) | 0.05 |  | 277 | 0.63 (0.10, 1.16) | 0.02 |  |
| Predialysis (Prior to study visit) | 105 | 0.53 (0.09, 0.97) | 0.02 |  | 274 | 0.13 (-0.20, 0.47) | 0.43 |  |
| Non-dialysis (Seated) | 106 | 0.61 (0.08, 1.14) | 0.03 |  | 281 | 0.69 (0.33, 1.05) | < 0.001 |  |
| Non-dialysis (Supine) | 103 | 0.38 (-0.13, 0.89) | 0.14 |  | 264 | 0.71 (0.34, 1.08) | < 0.001 |  |
|  |  |  |  |  |  |  |  |  |
| **Arterial stiffness measurements** |  |  |  |  |  |  |  |  |
| **Pulse wave velocity (m/s)** | 89 | -2.58 (-5.16, 0.01) | 0.05 |  | 248 | 1.36 (-0.64, 3.36) | 0.18 |  |
| **Central augmentation index** | 99 | 0.0008 (-0.60, 0.60) | 0.99 |  | 266 | -0.03 (-0.54, 0.47) | 0.90 |  |
|  |  |  |  |  |  |  |  |  |
| **Volume measurements** |  |  |  |  |  |  |  |  |
| **Tricuspid regurgitation (Non-dialysis)** | 58 | -1.04 (-2.36, 0.28) | 0.12 |  | 153 | 0.36 (-0.35, 1.07) | 0.32 |  |
| **Intradialytic weight gain (3 month average)** | 106 | -1.30 (-9.42, 6.82) | 0.75 |  | 277 | 7.52 (1.55, 13.48) | 0.01 |  |
| **Intradialytic weight gain (Prior to study visit)** | 105 | 3.77 (-3.12, 10.65) | 0.28 |  | 275 | 2.94 (0.04, 5.84) | 0.05 |  |
|  |  |  |  |  |  |  |  |  |
| *Adjusted model accounted for all of the following baseline covariates: age, sex, ethnicity, body mass index, history of hypercholesterolemia, smoking, diabetes mellitus, coronary heart disease, congestive heart failure, cause of end-stage renal disease, time since first nephrology visit, beta blocker use, RAAS blockade use, number of antihypertensive medication, and dietary sodium intake | | | | | | | | |
|  |  |  |  |  |  |  |  |  |

**Supplementary Table 6.** Association of preload and afterload measures with LVMI by linear regression among incident hemodialysis participants stratified by 3 month average IDWG groups

|  |  |  |  |  |  |  |  |  |  |  |  |
| --- | --- | --- | --- | --- | --- | --- | --- | --- | --- | --- | --- |
| **Variables** | **Adjusted* (IDWG 0-2kg)** | | |  | **Adjusted* (IDWG 2-3kg)** | | |  | **Adjusted* (IDWG >3kg)** | | |
|  | **n** | **β (95% CI)** | ***P*** |  | **n** | **β (95% CI)** | ***P*** |  | **n** | **β (95% CI)** | ***P*** |
|  |  |  |  |  |  |  |  |  |  |  |  |
| **Vascular measurements** |  |  |  |  |  |  |  |  |  |  |  |
| **Blood pressure (per 10 mmHg)** |  |  |  |  |  |  |  |  |  |  |  |
| **Predialysis (3 month average)** | 154 |  |  |  | 144 |  |  |  | 85 |  |  |
| Systolic |  | 6.05 (0.82, 11.27) | 0.02 |  |  | 6.58 (1.68, 11.48) | 0.009 |  |  | 5.71 (-1.09, 12.51) | 0.10 |
| Diastolic |  | 7.80 (-2.62, 18.22) | 0.14 |  |  | 9.05 (1.81, 16.28) | 0.02 |  |  | 7.43 (-4.14, 19.00) | 0.20 |
| **Predialysis (Prior to study visit)** | 154 |  |  |  | 140 |  |  |  | 84 |  |  |
| Systolic |  | 2.95 (-0.38, 6.27) | 0.08 |  |  | 3.19 (0.17, 6.22) | 0.04 |  |  | -1.84 (-6.32, 2.65) | 0.42 |
| Diastolic |  | 6.62 (0.67, 12.57) | 0.03 |  |  | 5.13 (-0.04, 10.31) | 0.05 |  |  | -2.38 (-10.03, 5.27) | 0.54 |
| **Non-dialysis (Seated)** | 154 |  |  |  | 141 |  |  |  | 84 |  |  |
| Systolic |  | 8.54 (5.17, 11.91) | < 0.001 |  |  | 2.54 (-0.46, 5.54) | 0.10 |  |  | 0.01 (-4.43, 4.45) | 0.99 |
| Diastolic |  | 13.95 (7.38, 20.53) | < 0.001 |  |  | 6.42 (0.81, 12.03) | 0.03 |  |  | -3.03 (-10.56, 4.49) | 0.42 |
| **Non-dialysis (Supine)** | 149 |  |  |  | 130 |  |  |  | 80 |  |  |
| Systolic |  | 6.64 (3.29, 9.98) | < 0.001 |  |  | 4.89 (1.40, 8.37) | 0.006 |  |  | 1.30 (-2.94, 5.53) | 0.54 |
| Diastolic |  | 10.38 (3.88, 16.88) | 0.002 |  |  | 7.88 (1.87, 13.89) | 0.01 |  |  | -0.60 (-8.39, 7.19) | 0.88 |
| **Mean arterial pressure** | 149 | 0.91 (0.44, 1.39) | < 0.001 |  | 134 | 0.65 (0.23, 1.06) | 0.002 |  | 80 | -0.0007 (-0.56, 0.56) | 0.99 |
| **Pulse pressure** |  |  |  |  |  |  |  |  |  |  |  |
| Predialysis (3 months average) | 154 | 0.68 (0.0007, 1.37) | 0.05 |  | 144 | 0.57 (-0.20, 1.34) | 0.15 |  | 85 | 0.66 (-0.34, 1.66) | 0.19 |
| Predialysis (Prior to study visit) | 153 | 0.29 (-0.16, 0.74) | 0.21 |  | 140 | 0.30 (-0.14, 0.73) | 0.18 |  | 84 | -0.23 (-0.90, 0.45) | 0.50 |
| Non-dialysis (Seated) | 154 | 0.96 (0.46, 1.46) | < 0.001 |  | 141 | 0.16 (-0.29, 0.61) | 0.47 |  | 84 | 0.33 (-0.45, 1.10) | 0.41 |
| Non-dialysis (Supine) | 149 | 0.81 (0.31, 1.30) | 0.002 |  | 130 | 0.48 (-0.04, 1.00) | 0.07 |  | 80 | 0.37 (-0.30, 1.04) | 0.27 |
|  |  |  |  |  |  |  |  |  |  |  |  |
| **Arterial stiffness measurements** |  |  |  |  |  |  |  |  |  |  |  |
| **Pulse wave velocity (m/s)** | 137 | 1.39 (-1.30, 4.09) | 0.31 |  | 123 | 2.64 (-0.25, 5.54) | 0.07 |  | 70 | -1.84 (-5.58, 1.91) | 0.33 |
| **Central augmentation index** | 145 | -0.44 (-1.12, 0.24) | 0.20 |  | 133 | 0.68 (0.02, 1.35) | 0.04 |  | 80 | -0.24 (-0.94, 0.47) | 0.50 |
|  |  |  |  |  |  |  |  |  |  |  |  |
| **Volume measurements** |  |  |  |  |  |  |  |  |  |  |  |
| **Tricuspid regurgitation (Non-dialysis)** | 87 | 0.01 (-1.02, 1.04) | 0.98 |  | 79 | 0.30 (-0.90, 1.49) | 0.62 |  | 41 | -1.15 (-3.22, 0.93) | 0.26 |
|  |  |  |  |  |  |  |  |  |  |  |  |
| *Adjusted model accounted for all of the following baseline covariates: age, sex, ethnicity, body mass index, history of hypercholesterolemia, smoking, diabetes mellitus, coronary heart disease, congestive heart failure, cause of end-stage renal disease, time since first nephrology visit, beta blocker use, RAAS blockade use, number of antihypertensive medication, and dietary sodium intake | | | | | | | | | | | |
|  |  |  |  |  |  |  |  |  |  |  |  |

**Supplementary Table 7.** Association of preload and afterload measures with LVMI by linear regression among incident hemodialysis participants stratified by β-blocker medication

|  |  |  |  |  |  |  |  |
| --- | --- | --- | --- | --- | --- | --- | --- |
| **Variables** |  | **Adjusted* (No beta-blocker)** | |  |  | **Adjusted* (Beta-blocker)** | |
|  | **n** | **β (95% CI)** | **P value** |  | **n** | **β (95% CI)** | **P value** |
|  |  |  |  |  |  |  |  |
| **Vascular measurements** |  |  |  |  |  |  |  |
| **Blood pressure (per 10 mmHg)** |  |  |  |  |  |  |  |
| **Predialysis (3 month average)** | 92 |  |  |  | 237 |  |  |
| Systolic |  | 9.07 (1.65, 16.49) | 0.02 |  |  | 6.13 (2.40, 9.85) | 0.001 |
| Diastolic |  | 4.40 (-7.85, 16.65) | 0.48 |  |  | 11.78 (5.39, 18.18) | < 0.001 |
| **Predialysis (Prior to study visit)** | 90 |  |  |  | 238 |  |  |
| Systolic |  | 3.12 (-1.43, 7.66) | 0.18 |  |  | 1.41 (-1.11, 3.93) | 0.27 |
| Diastolic |  | 1.04 (-6.20, 8.29) | 0.78 |  |  | 5.04 (0.52, 9.56) | 0.03 |
| **Non-dialysis (Seated)** | 93 |  |  |  | 241 |  |  |
| Systolic |  | 4.77 (-0.24, 9.78) | 0.06 |  |  | 4.90 (2.51, 7.28) | < 0.001 |
| Diastolic |  | 3.88 (-4.04, 11.8) | 0.33 |  |  | 9.07 (4.53, 13.6) | < 0.001 |
| **Non-dialysis (Supine)** | 88 |  |  |  | 230 |  |  |
| Systolic |  | 5.95 (0.70, 11.20) | 0.03 |  |  | 4.09 (1.62, 6.56) | 0.001 |
| Diastolic |  | 4.91 (-3.01, 12.83) | 0.22 |  |  | 7.57 (2.90, 12.25) | 0.002 |
| **Mean arterial pressure (Non-dialysis)** | 89 | 0.58 (-0.08, 1.24) | 0.09 |  | 231 | 0.59 (0.25, 0.93) | 0.001 |
| **Pulse pressure** |  |  |  |  |  |  |  |
| Predialysis (3 months average) | 92 | 1.26 (0.29, 2.23) | 0.01 |  | 237 | 0.42 (-0.11, 0.95) | 0.12 |
| Predialysis (Prior to study visit) | 90 | 0.57 (-0.08, 1.23) | 0.09 |  | 238 | 0.04 (-0.29, 0.37) | 0.79 |
| Non-dialysis (Seated) | 93 | 0.97 (0.10, 1.84) | 0.03 |  | 241 | 0.50 (0.15, 0.86) | 0.005 |
| Non-dialysis (Supine) | 88 | 0.86 (0.06, 1.67) | 0.04 |  | 230 | 0.41 (0.05, 0.78) | 0.03 |
|  |  |  |  |  |  |  |  |
| **Arterial stiffness measurements** |  |  |  |  |  |  |  |
| **Pulse wave velocity (m/s)** | 79 | 1.42 (-2.79, 5.62) | 0.50 |  | 207 | 0.10 (-1.84, 2.04) | 0.92 |
| **Central augmentation index** | 89 | 0.28 (-0.67, 1.23) | 0.56 |  | 226 | -0.23 (-0.71, 0.26) | 0.36 |
|  |  |  |  |  |  |  |  |
| **Volume measurements** |  |  |  |  |  |  |  |
| **Tricuspid regurgitation (Non-dialysis)** | 55 | 0.35 (-0.86, 1.56) | 0.56 |  | 123 | 0.02 (-0.89, 0.93) | 0.97 |
| **Intradialytic weight gain (3 month average)** | 92 | 9.60 (-0.20, 19.40) | 0.06 |  | 237 | 1.95 (-4.14, 8.03) | 0.53 |
| **Intradialytic weight gain (Prior to study visit)** | 90 | 11.95 (4.67, 19.22) | 0.002 |  | 238 | 1.45 (-1.62, 4.53) | 0.35 |
|  |  |  |  |  |  |  |  |
| *Adjusted model accounted for all of the following baseline covariates: age, sex, ethnicity, body mass index, history of hypercholesterolemia, smoking, diabetes mellitus, coronary heart disease, congestive heart failure, cause of end-stage renal disease, time since first nephrology visit, beta blocker use, RAAS blockade use, number of antihypertensive medication, and dietary sodium intake | | | | | | | |

**Supplementary Table 8.** Association of preload and afterload measures with LVMI by linear regression among incident hemodialysis participants stratified by renin-angiotensin-aldosterone system (RAAS) blockade* use

|  |  |  |  |  |  |  |  |
| --- | --- | --- | --- | --- | --- | --- | --- |
| **Variables** |  | **Adjusted* (No RAAS blockade)** | |  |  | **Adjusted** (RAAS blockade)** | |
|  | **n** | **β (95% CI)** | **P value** |  | **n** | **β (95% CI)** | **P value** |
|  |  |  |  |  |  |  |  |
| **Vascular measurements** |  |  |  |  |  |  |  |
| **Blood pressure (per 10 mmHg)** |  |  |  |  |  |  |  |
| **Predialysis (3 month average)** | 177 |  |  |  | 152 |  |  |
| Systolic |  | 6.88 (2.88, 10.88) | 0.001 |  |  | 7.03 (1.61, 12.45) | 0.01 |
| Diastolic |  | 7.59 (0.57, 14.61) | 0.03 |  |  | 12.72 (3.84, 21.61) | 0.005 |
| **Predialysis (Prior to study visit)** | 176 |  |  |  | 152 |  |  |
| Systolic |  | 2.41 (-0.15, 4.98) | 0.07 |  |  | 0.99 (-2.47, 4.46) | 0.57 |
| Diastolic |  | 4.24 (-0.54, 9.02) | 0.08 |  |  | 1.80 (-4.00, 7.60) | 0.54 |
| **Non-dialysis (Seated)** | 180 |  |  |  | 154 |  |  |
| Systolic |  | 5.01 (2.44, 7.57) | < 0.001 |  |  | 2.98 (-0.53, 6.50) | 0.10 |
| Diastolic |  | 6.71 (1.89, 11.52) | 0.007 |  |  | 6.31 (-0.07, 12.69) | 0.05 |
| **Non-dialysis (Supine)** | 172 |  |  |  | 146 |  |  |
| Systolic |  | 4.81 (1.94, 7.68) | 0.001 |  |  | 2.98 (-0.49, 6.44) | 0.09 |
| Diastolic |  | 6.22 (0.95, 11.48) | 0.02 |  |  | 6.74 (0.46, 13.02) | 0.04 |
| **Mean arterial pressure (Non-dialysis)** | 173 | 0.54 (0.15, 0.93) | 0.007 |  | 147 | 0.46 (0.0009, 0.91) | 0.05 |
| **Pulse pressure** |  |  |  |  |  |  |  |
| Predialysis (3 months average) | 177 | 0.82 (0.26, 1.38) | 0.004 |  | 152 | 0.43 (-0.32, 1.18) | 0.26 |
| Predialysis (Prior to study visit) | 176 | 0.24 (-0.10, 0.57) | 0.16 |  | 152 | 0.07 (-0.41, 0.54) | 0.78 |
| Non-dialysis (Seated) | 180 | 0.70 (0.30, 1.09) | 0.001 |  | 154 | 0.24 (-0.29, 0.77) | 0.37 |
| Non-dialysis (Supine) | 172 | 0.60 (0.18, 1.02) | 0.005 |  | 146 | 0.22 (-0.31, 0.75) | 0.42 |
|  |  |  |  |  |  |  |  |
| **Arterial stiffness measurements** |  |  |  |  |  |  |  |
| **Pulse wave velocity (m/s)** | 155 | 0.45 (-1.55, 2.46) | 0.66 |  | 131 | -0.11 (-2.97, 2.75) | 0.94 |
| **Central augmentation index** | 171 | -0.04 (-0.55, 0.47) | 0.88 |  | 144 | -0.54 (-1.27, 0.20) | 0.15 |
|  |  |  |  |  |  |  |  |
| **Volume measurements** |  |  |  |  |  |  |  |
| **Tricuspid regurgitation (Non-dialysis)** | 90 | -0.16 (-1.32, 1.00) | 0.78 |  | 88 | 0.21 (-0.63, 1.05) | 0.62 |
| **Intradialytic weight gain (3 month average)** | 177 | 3.52 (-2.69, 9.72) | 0.27 |  | 152 | 6.40 (-2.31, 15.12) | 0.15 |
| **Intradialytic weight gain (Prior to study visit)** | 176 | 1.72 (-1.92, 5.35) | 0.35 |  | 152 | 3.64 (-0.55, 7.83) | 0.09 |
|  |  |  |  |  |  |  |  |
| * Renin-angiotensin-aldosterone system blockade medications include angiotensin-converting-enzyme inhibitor and angiotensin II receptor blocker  **Adjusted model accounted for all of the following baseline covariates: age, sex, ethnicity, body mass index, history of hypercholesterolemia, smoking, diabetes mellitus, coronary heart disease, congestive heart failure, cause of end-stage renal disease, time since first nephrology visit, beta blocker use, RAAS blockade use, number of antihypertensive medication, and dietary sodium intake | | | | | | | |

**Supplementary Table 9.** Association of preload and afterload measures with LVMI by linear regression among incident hemodialysis participants stratified by history of congestive heart failure

|  |  |  |  |  |  |  |  |
| --- | --- | --- | --- | --- | --- | --- | --- |
| **Variables** | **Adjusted* (No congestive heart failure)** | | |  | **Adjusted* (Congestive heart failure)** | | |
|  | **n** | **β (95% CI)** | **P value** |  | **n** | **β (95% CI)** | **P value** |
|  |  |  |  |  |  |  |  |
| **Vascular measurements** |  |  |  |  |  |  |  |
| **Blood pressure (per 10 mmHg)** |  |  |  |  |  |  |  |
| **Predialysis (3 month average)** | 227 |  |  |  | 156 |  |  |
| Systolic |  | 7.89 (4.22, 11.55) | < 0.001 |  |  | 5.90 (0.33, 11.46) | 0.04 |
| Diastolic |  | 11.75 (5.94, 17.56) | < 0.001 |  |  | 8.3 (-1.79, 18.38) | 0.11 |
| **Predialysis (Prior to study visit)** | 225 |  |  |  | 155 |  |  |
| Systolic |  | 3.69 (1.48, 5.91) | 0.001 |  |  | -0.61 (-4.24, 3.03) | 0.74 |
| Diastolic |  | 5.61 (1.87, 9.35) | 0.003 |  |  | -0.42 (-7.12, 6.28) | 0.90 |
| **Non-dialysis (Seated)** | 229 |  |  |  | 158 |  |  |
| Systolic |  | 5.14 (2.66, 7.62) | < 0.001 |  |  | 4.22 (0.74, 7.69) | 0.02 |
| Diastolic |  | 7.20 (2.90, 11.49) | 0.001 |  |  | 6.90 (-0.07, 13.87) | 0.05 |
| **Non-dialysis (Supine)** | 217 |  |  |  | 150 |  |  |
| Systolic |  | 5.37 (2.83, 7.92) | < 0.001 |  |  | 3.28 (-0.39, 6.94) | 0.08 |
| Diastolic |  | 7.37 (3.04, 11.70) | 0.001 |  |  | 3.23 (-3.97, 10.43) | 0.38 |
| **Mean arterial pressure (Non-dialysis)** | 222 | 0.61 (0.29, 0.93) | < 0.001 |  | 148 | 0.39 (-0.12, 0.90) | 0.13 |
| **Pulse pressure** |  |  |  |  |  |  |  |
| Predialysis (3 months average) | 227 | 0.71 (0.15, 1.28) | 0.01 |  | 156 | 0.52 (-0.18, 1.22) | 0.15 |
| Predialysis (Prior to study visit) | 225 | 0.34 (0.02, 0.66) | 0.04 |  | 155 | 0.04 (-0.47, 0.55) | 0.87 |
| Non-dialysis (Seated) | 229 | 0.65 (0.26, 1.05) | 0.001 |  | 158 | 0.54 (0.02, 1.05) | 0.04 |
| Non-dialysis (Supine) | 217 | 0.61 (0.22, 0.99) | 0.002 |  | 150 | 0.50 (-0.02, 1.03) | 0.06 |
|  |  |  |  |  |  |  |  |
| **Arterial stiffness measurements** |  |  |  |  |  |  |  |
| **Pulse wave velocity (m/s)** | 200 | 1.80 (-0.32, 3.92) | 0.10 |  | 137 | -2.32 (-5.04, 0.40) | 0.09 |
| **Central augmentation index** | 219 | 0.08 (-0.44, 0.59) | 0.77 |  | 146 | -0.29 (-0.95, 0.37) | 0.38 |
|  |  |  |  |  |  |  |  |
| **Volume measurements** |  |  |  |  |  |  |  |
| **Tricuspid regurgitation (Non-dialysis)** | 125 | -0.05 (-0.89, 0.78) | 0.90 |  | 86 | 0.21 (-0.92, 1.34) | 0.72 |
| **Intradialytic weight gain (3 month average)** | 227 | 0.41 (-5.74, 6.56) | 0.90 |  | 156 | 9.17 (1.55, 16.79) | 0.02 |
| **Intradialytic weight gain (Prior to study visit)** | 225 | 1.89 (-1.16, 4.94) | 0.22 |  | 155 | 5.93 (1.08, 10.77) | 0.02 |
|  |  |  |  |  |  |  |  |
| *Adjusted model accounted for all of the following baseline covariates: age, sex, ethnicity, body mass index, history of hypercholesterolemia, smoking, diabetes mellitus, coronary heart disease, congestive heart failure, cause of end-stage renal disease, time since first nephrology visit, beta blocker use, RAAS blockade use, number of antihypertensive medication, and dietary sodium intake | | | | | | | |

**Supplementary Table 10.** Association of hemoglobin with LVMI by linear regression among incident hemodialysis participants

|  |  |  |  |  |  |  |
| --- | --- | --- | --- | --- | --- | --- |
| **Variables** | **n** | **Unadjusted** | |  | **Adjusted*** | |
|  |  | **β (95% CI)** | ***P*** |  | **β (95% CI)** | ***P*** |
|  |  |  |  |  |  |  |
| Hemoglobin (3 month average) | 361 | -3.18 (-7.43, 1.06) | 0.14 |  | -2.61 (-6.83, 1.60) | 0.22 |
|  |  |  |  |  |  |  |
| *Adjusted model accounted for all of the following baseline covariates: age, sex, ethnicity, body mass index, history of hypercholesterolemia, smoking, diabetes mellitus, coronary heart disease, congestive heart failure, cause of end-stage renal disease, time since first nephrology visit, beta blocker use, RAAS blockade use, number of antihypertensive medication, and dietary sodium intake | | | | | | |
